# Supplementary material for: Modulation of M2 macrophage polarization by the crosstalk between Stat6 and Trim24
Source: Nat Commun. 2019 Sep 25;10:4353. doi: 10.1038/s41467-019-12384-2 (PMC6761150; doi:10.1038/s41467-019-12384-2)
Supplement: Supplementary file 1 — Supplementary Information [file 41467_2019_12384_MOESM1_ESM.pdf]

Supplementary Information

STAT6 acetylation curtails macrophage M2 polarization and potentiates anti-tumor immunity

by Yu *et al*.

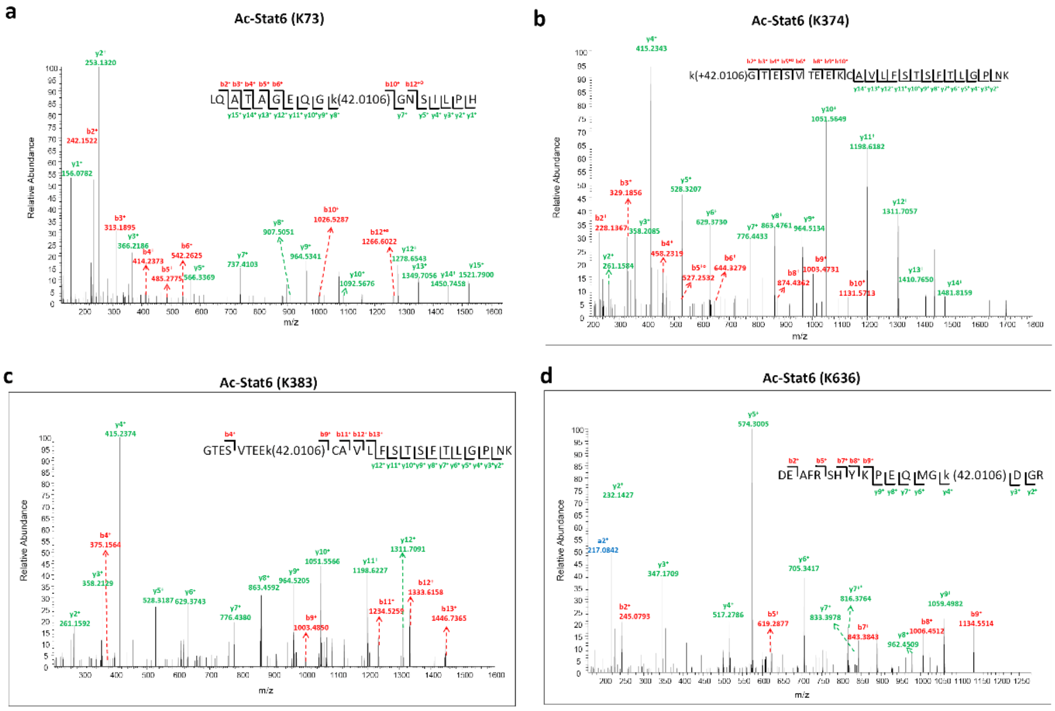

Supplementary Figure 1. Mass spectroscopy showing the potential acetylation sites of Stat6 after immunoprecipitation of Stat6 in 293T cells transfected with Stat6 and CBP.

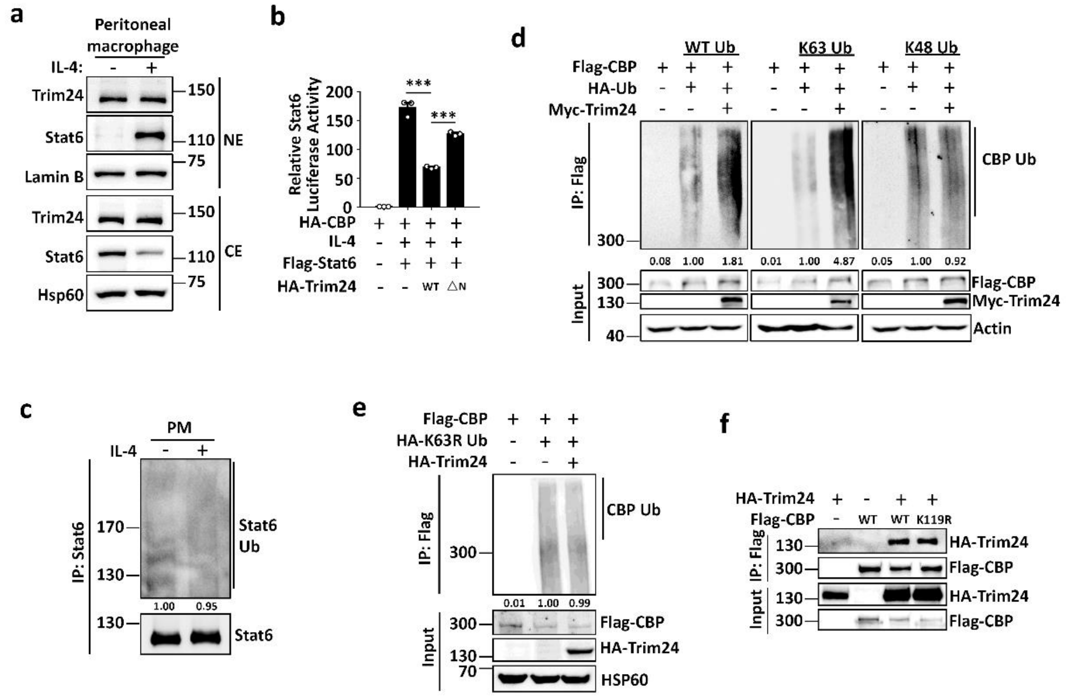

Supplementary Figure 2. Trim24 mediated Lys63-linked ubiquitination of CBP

(**a**) Immunoblot of Trim24, Stat6 and Lamin B or Hsp60 in nuclear and cytosol extracts of peritoneal macrophages that stimulated with (+) or without (-) IL-4. (**b**) Luciferase assay of Stat6 transcriptional activity in 293T cells that transfected with the indicated expression vectors, then were stimulated with IL-4 for 4 h before detection. (**c**) Immunoblot of endogenous Stat6 ubiquitination in murine primary macrophages that were left unstimulated (-) or stimulated (+) with IL-4 for 30 min. (**d** and **e**) Ubiquitination of CBP in 293T cells transfected with the indicated expression vectors, assessed by immunoblot analysis with anti-HA after immunoprecipitation with anti-Flag (top) or by immunoblot analysis with input proteins in lysates without immunoprecipitation (below). (**f**) Co-immunoprecipitation analysis of the binding between Trim24 and WT or K119R mutant CBP in the 293T cells transfected with the indicated expression vectors, assessed by immunoprecipitation (IP) with anti-Flag and immunoblot with anti-HA and anti-Flag. Data with error bars are represented as mean ± SD. Each panel is a representative experiment of at least three independent biological replicates. ∗∗$p < 0.01$ as determined by unpaired Student's t test. Source data are provided as a Source Data file.

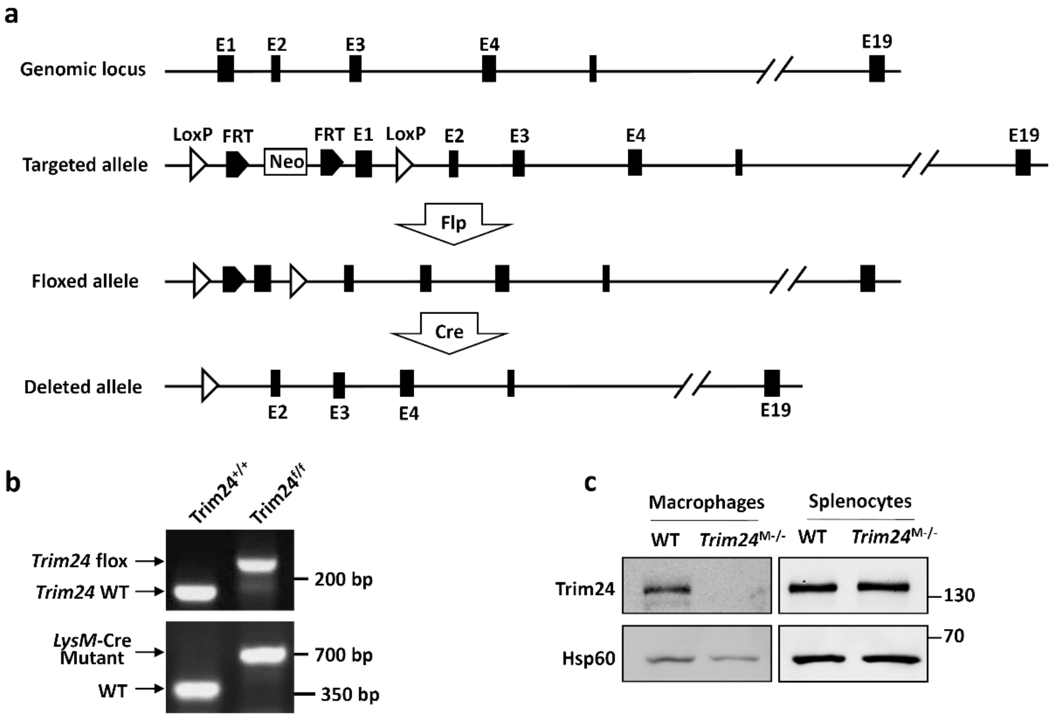

Supplementary Figure 3. Scheme of the construction of Trim24 floxed mouse and verification of Trim24 deficiency in macrophages.

(**a**) Schematic picture of Trim24 gene targeting using an FRT-LoxP vector. Targeted mice were crossed with FRT deleter (Rosa26-FLPe) mice to generate Trim24-floxed mice, which were further crossed with Lysosome M (LysM) Cre mice to generate myeloid cells conditional knockout mice. (**b**) Genotyping PCR analysis of Trim24$^{+/+}$LysM-cre^{-} and Trim24$^{f/f}$LysM-cre^{+} mice. (**c**) Immunoblot analysis of Trim24 and Hsp60 (loading control) protein expression in macrophages and splenocytes that isolated from wild-type (WT) and *Trim24*$^{M-/-}$ mice. Source data are provided as a Source Data file.

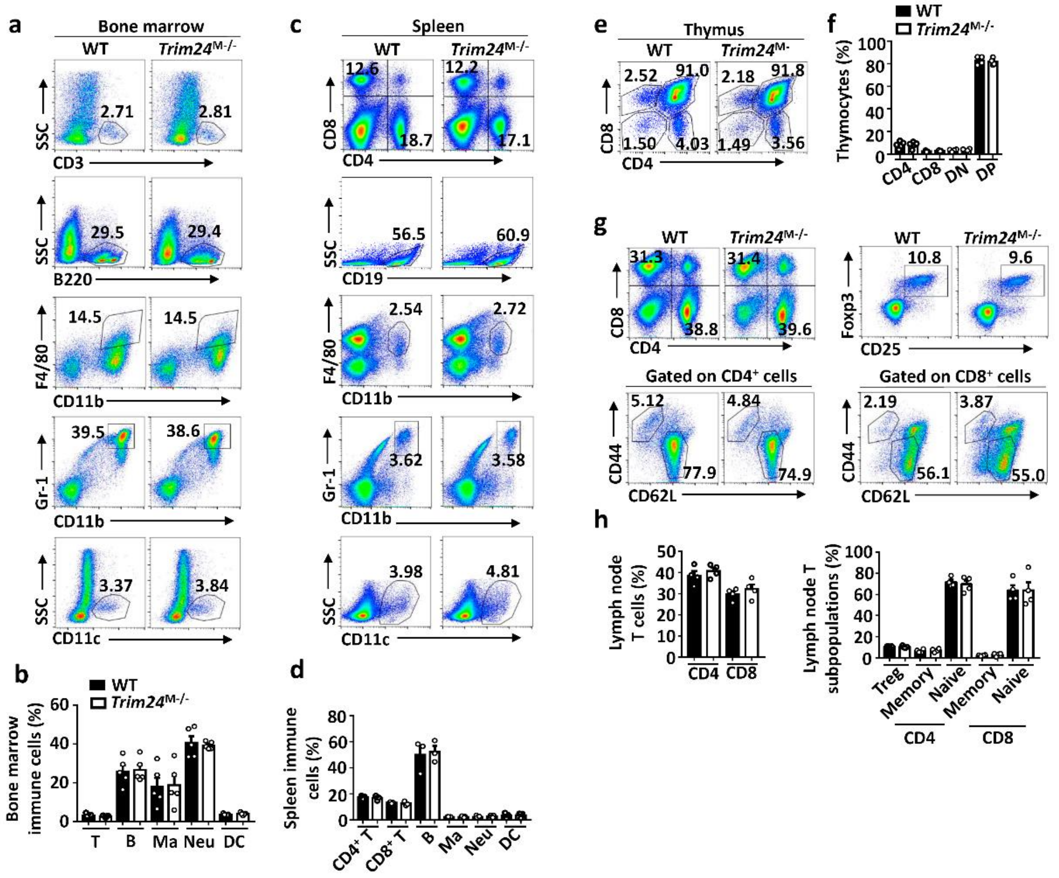

Supplementary Figure 4. Trim24 does not affect the development, maturation and activation of myeloid and lymphoid cells.

(**a-h**) Flow cytometry of the frequencies of the indicated lymphoid and myeloid immune cells in the bone marrow (**a** and **b**), spleen (**c** and **d**), thymus (**e** and **f**), and peripheral lymph nodes (**g** and **h**) of wild-type (WT) and *Trim24*$^{M-/-}$ mice. Data are presented as representative plots (**a, c, e** and **g**) and summary bar graph (**b, d, f** and **h**). Data are representative of three independent experiments. Data with error bars are represented as mean ± SD. Each panel is a representative experiment of at least three independent biological replicates. Source data are provided as a Source Data file.

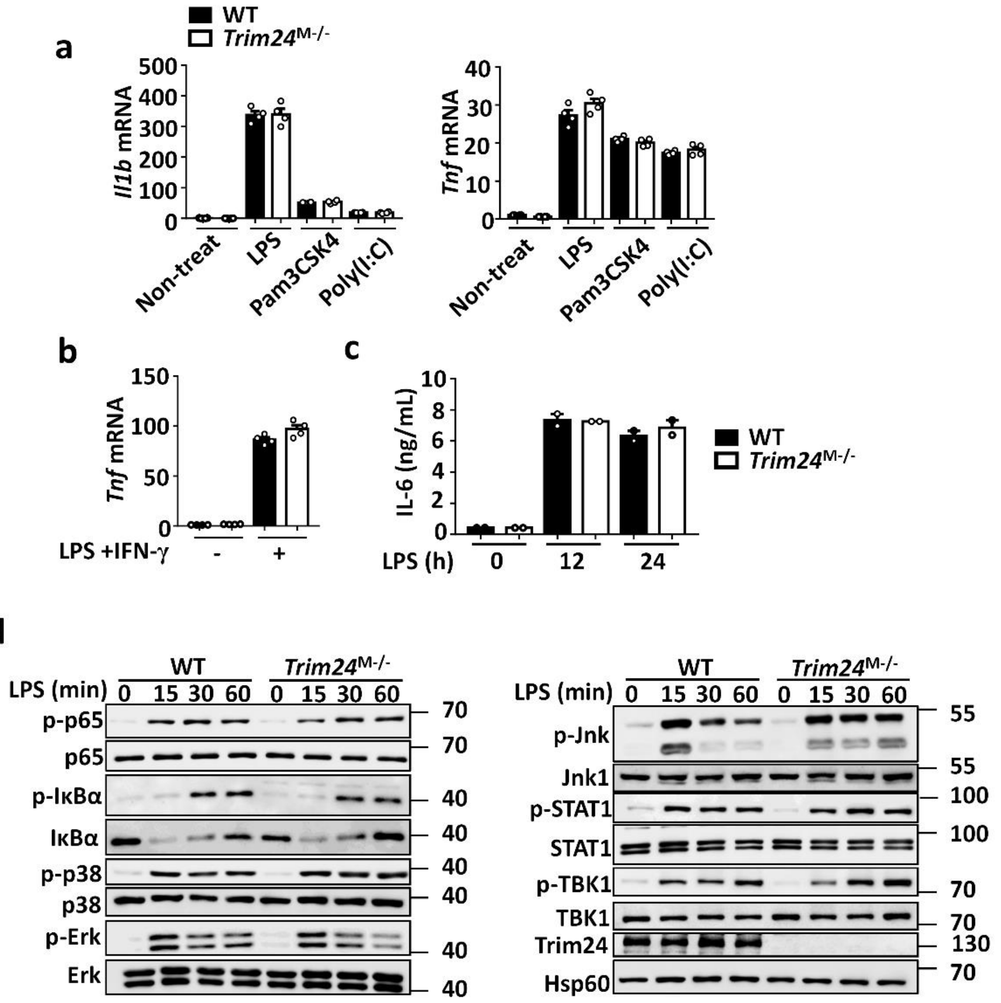

Supplementary Figure 5. Trim24 does not affect macrophage M1 polarization.

(**a** and **b**) QPCR analysis of *Il1b* and *Tnf* mRNA level in wild-type (WT) and *Trim24*-deficient (*Trim24*$^{M-/-}$) macrophage that were left non-treated or stimulated with TLR ligands LPS (100 ng/ml), Pam3CSK4 (100 ng/ml) and Poly (I:C) (20 μg/ml) or with LPS plus IFNγ (100 ng/ml). (**c**) ELISA showing the secretion of IL-6 in supernatants of WT and *Trim24*-deficient macrophage that were stimulated with LPS at the indicated time. (**d**) Immunoblot analysis of phosphorylated (P-) and total NF-κB, MAPKs, TBK1 and Stat1 signaling proteins, Trim24, or Hsp60 (loading control) in whole-cell lysates of WT and *Trim24*-deficient macrophages that were left unstimulated or stimulated with LPS (100 ng/ml) at the indicated time. Data with error bars are represented as mean ± SD. Each panel is a representative experiment of at least three independent biological replicates. Source data are provided as a Source Data file.

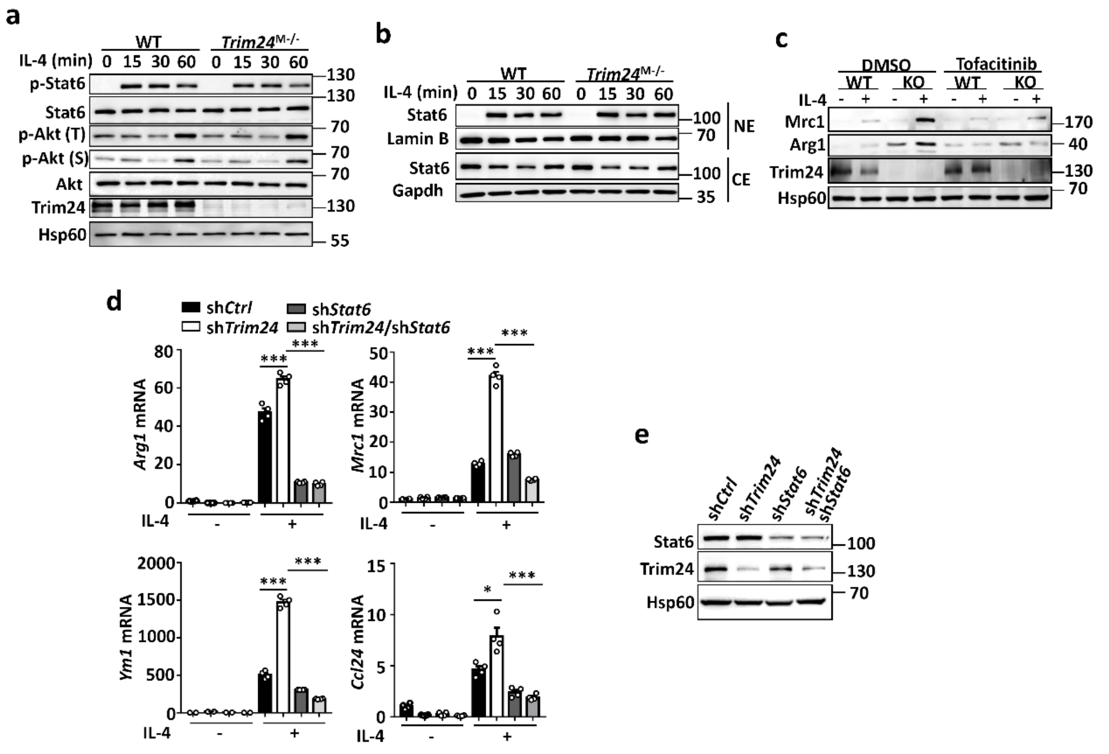

Supplementary Figure 6. STAT6 is required for the enhanced M2 gene induction in *Trim24*-deficient macrophages.

(**a** and **b**) Immunoblot analysis of phosphorylated (P-) and total Stat6 and Akt, Trim24, or Hsp60 (loading control) in whole-cell lysates (**a**) and immunoblot of Stat6, Lamin B or Gapdh in nuclear and cytoplasmic extracts (**b**) of WT and *Trim24*-deficient macrophages that were stimulated with IL-4 (20 ng/ml) for indicated time. (**c**) Immunoblot of Mrc1, Arg1, Trim24 and Hsp60 in WT and *Trim24*-deficient macrophages that pretreated with DMSO or Jak3 selective inhibitor tofacitinib, and then stimulated with (+) or without (-) IL-4 (20 ng/ml) for 48 h. (**d** and **e**) QPCR analysis of IL-4-induced *Arg1*, *Mrc1*, *Ym1* and *Ccl24* mRNA expression in immortalized BMDMs (iBMDMs) upon knockdown of *Trim24* or *Stat6* or *Trim24*/*Stat6* double knockdown (**d**). The knockdown efficiency of Stat6 or Trim24 in iBMDMs are showed by immunoblot analysis in (**e**). Data with error bars are represented as mean ± SD. Each panel is a representative experiment of at least three independent biological replicates. $*p < 0.05$, $**p < 0.01$, $***p < 0.001$ as determined by unpaired Student's t test. Source data are provided as a Source Data file.

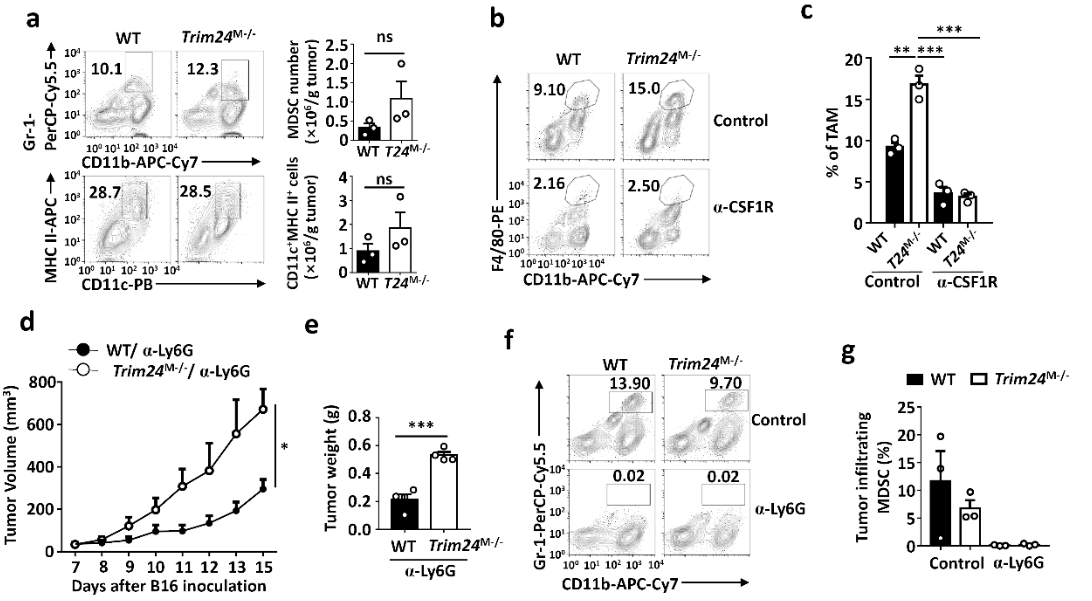

Supplementary Figure 7. MDSC is dispensable for the tumor growth in *Trim24*$^{M-/-}$ mice. (**a**) Flow cytometry analysis of the frequencies and absolute numbers of tumor-infiltrating $CD45^+CD11b^+Gr\text{-}1^+$ MDSC and $CD45^+CD11c^+MHC\ II^+$ dendritic cells in B16 melanoma-bearing wild-type (WT) and *Trim24*$^{M-/-}$ mice. Data are presented as representative plots (left panels) and summary bar graphs showing absolute cell numbers (right panels). (**b, c**) Flow cytometric analysis showing the depletion efficiency of $CD45^+CD11b^+F4/80^+$ macrophages in B16 melanoma of WT and *Trim24*$^{M-/-}$ mice. Data are presented as representative plots (**b**) and a statistical bar graph (**c**). (**d** and **e**) Tumor growth curve (**d**) and weight (**e**) of wild-type (WT) and *Trim24*$^{M-/-}$ mice that were intravenously injected with anti-Ly6G antibody at a 3-day interval for 4 times starting from the one day before tumor inoculation, and then challenged with B16 tumor cells subcutaneously. (**f** and **g**) Flow cytometric analysis showing the depletion efficiency of MDSC in B16 melanoma of WT and *Trim24*$^{M-/-}$ mice. Data are presented as a representative plot (**f**) and summary graph (**g**). Data with error bars are represented as mean ± SD. Each panel is a representative experiment of at least three independent biological replicates. *p < 0.05, **p < 0.01, ***p < 0.001 as determined by unpaired Student's t test. Source data are provided as a Source Data file.

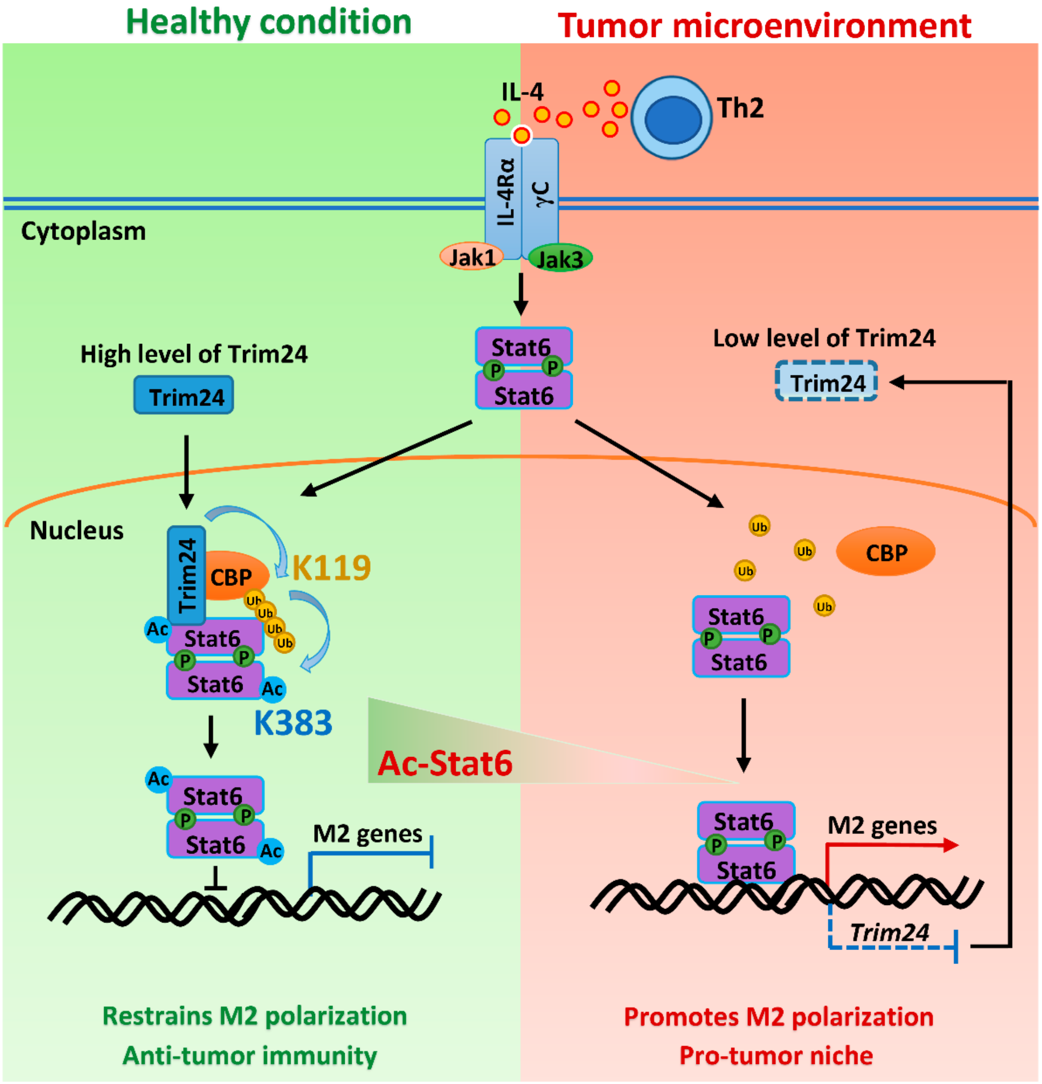

Supplementary Figure 8. The working model of Stat6 acetylation in regulating M2 polarization and anti-tumor response of macrophage.

Under healthy condition, Th2 cytokine IL-4 could induce the phosphorylation of Stat6, which were then translocated into the nucleus. Trim24 in nucleus was associated with CBP and mediated IL-4-induced Lys63-linked ubiquitination of CBP at Lys119. The ubiquitinated CBP promoted the recruitment of Stat6 for its acetylation at Lys383, which inhibited its DNA-binding activity, and thus restrains macrophage M2 polarization and potentiate anti-tumor immunity. However in tumor microenvironment, activated Stat6 directly bond into the promoter region, and mediated the transcriptional suppression of *Trim24* gene. Accordingly, decreased Trim24 failed to induce CBP ubiquitination, and thus impaired Stat6 acetylation, which in turn promoted TAM M2 polarization, suppressed anti-tumor immune function, and contributed to the immunosuppressive tumor niche.

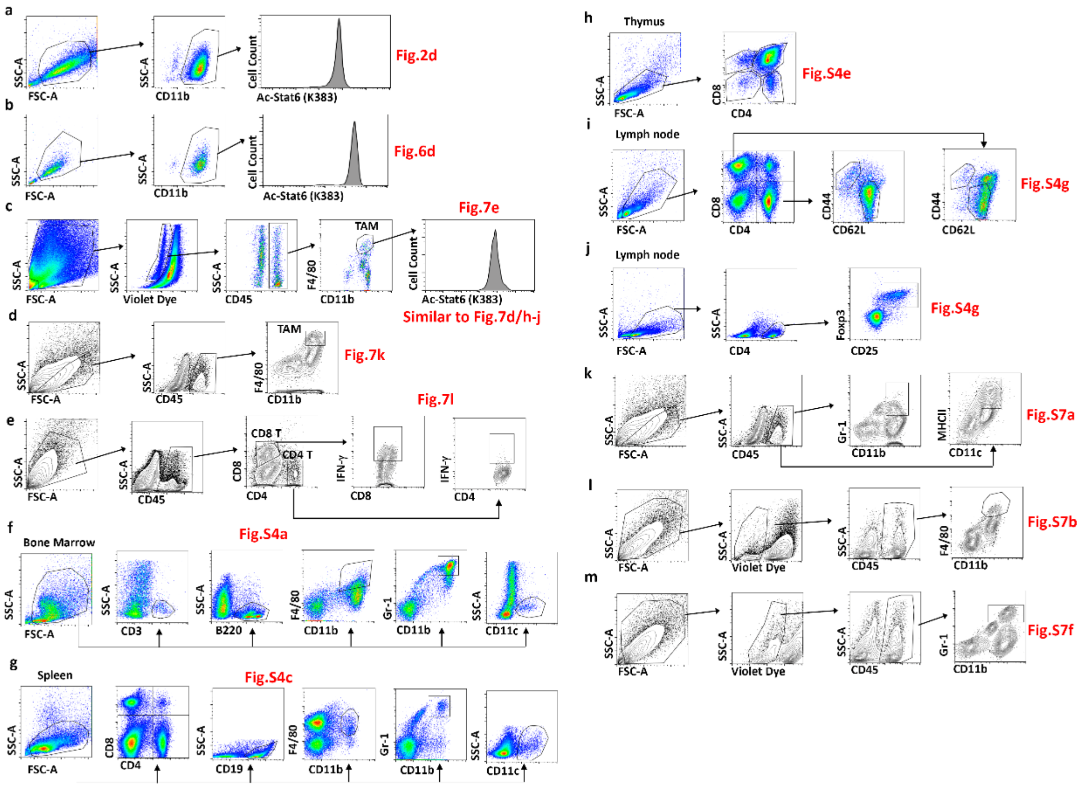

Supplementary Figure 9. Gating strategies used for flow cytometric analysis or cell sorting. (**a**) Flow cytometric strategy to detect endogenous Stat6 acetylation at Lys383 in murine primary peritoneal macrophages (CD11b^{+}) isolated from naïve C57BL/6 mice presented on Fig. 2d. (**b**) Flow cytometric strategy to detect endogenous Stat6 acetylation at Lys383 in control or *Trim24* knockdown human PBMC-derived primary macrophages (CD11b^{+}) presented on Fig. 6d. (**c**) Flow cytometric strategy of endogenous Stat6 acetylation detection at Lys383 in murine tumor-associated macrophage (TAM, CD45^{+}CD11b^{+}F4/80^{+}) from B16 melanoma of WT and *Trim24*$^{M-/-}$ mice presented on Fig. 7e. The gating strategies used for sorting TAM in Fig. 7d and Fig. 7h-7j is the same as (**c**). (**d-e**) Flow cytometric strategy to analysis the frequencies of TAM (CD45^{+}CD11b^{+}F4/80^{+}, Fig. 7k) and IFNγ-producing CD4^{+} (CD45^{+}CD4^{+}IFNγ^{+}, Fig. 7l) and CD8^{+} (CD45^{+}CD8^{+}IFNγ^{+}, Fig. 7l) T cells in B16 melanoma of WT and *Trim24*$^{M-/-}$ mice. (**f-j**) Flow cytometric strategy to analysis the frequencies of the indicated lymphoid and myeloid immune cells in the bone marrow (**f**), spleen (**g**), thymus (**h**) and peripheral lymph nodes (**I, j**) of WT and *Trim24*$^{M-/-}$ mice present on Supplementary Fig. 4. (**k**) Flow cytometric strategy to analysis the frequencies of the tumor-infiltrating MDSC (CD45^{+}CD11b^{+}Gr-1^{+}) and dendritic cells (CD45^{+}CD11c^{+}MHCII^{+}) in B16 melanoma of WT and *Trim24*$^{M-/-}$ mice presented on supplementary Fig. 7a. (**l**) Flow cytometric strategy to analysis the frequencies of TAM (CD45^{+}CD11b^{+}F4/80^{+}) in B16 melanoma of WT and *Trim24*$^{M-/-}$ mice presented on supplementary Fig. 7b. (**m**) Flow cytometric strategy to analysis the frequencies of tumor-infiltrating MDSC (CD45^{+}CD11b^{+}Gr-1^{+}) in B16 melanoma of WT and *Trim24*$^{M-/-}$ mice presented on supplementary Fig. 7f.

Supplementary Table 1. Primers used for real-time quantitative PCR

Genes	Forward primers (5'-3')	Reverse primers (5'-3')
m*Actb*	CGTGAAAAGATGACCCAGATCA	CACAGCCTGGATGGCTACGT
m*Il1b*	AAGCCTCGTGCTGTCGGACC	TGAGGCCCAAGGCCACAGGT
m*Tnf*	CATCTTCTCAAAATTCGAGTGACAA	CCAGCTGCTCCTCCACTTG
m*Arg1*	TTTTTCCAGCAGACCAGCTT	AGAGATTATCGGAGCGCCTT
m*Mrc1*	CAGGTGTGGGCTCAGGTAGT	TGGCATGTCCTGGAATGAT
m*Ym1*	TTTCTCCAGTGTAGCCATCCTT	TCTGGGTACAAGATCCCTGAA
m*Fizz1*	GGGATGACTGCTACTGGGTG	TCAACGAGTAAGCACAGGCA
m*Ccl24*	ATTCTGTGACCATCCCCTCAT	TGTATGTGCCTCTGAACCCAC
m*Irf4*	GCCCAACAAGCTAGAAAG	TCTCTGAGGGTCTGGAAACT
m*Trim24*	GCAAGCGGCTGATTACATACA	TGGTCACAGGAGGAAGCATCAC
h*ACTB*	ACTCTTCCAGCCTTCCTTCC	CGTACAGGTCTTTGCGGATG
h*TRIM24*	CCAGAACATACCACGACAAGCAA	GGAGTAGAGGATGTGCTGTTGG
h*TGM2*	TGTGGCACCAAGTACCTGCTCA	GCACCTTGATGAGGTTGGACTC
h*CCL17*	TTCTCTGCAGCACATCCACGCA	CTGGAGCAGTCCTCAGATGTCT
h*IRF4*	GAACGAGGAGAAGAGCATCTTCC	CGATGCCTTCTCGGAACTTTCC
m*Arg1* (ChIP)	GGCACAACTCACGTACAGACA	TGAGGCATTGTTCAGACTTCC
m*Ym1* (ChIP)	GTTCTTTGGAACTCTTAGGA	GCACACACCATTCTTCAGAA
m*Irf4* (ChIP)	GATCAAAGCAGAACAAGGCACAGACT	GGGAATCTACTTCCCAGACTCTTACA
h*TRIM24* (ChIP)	GGTTTATTTTTGCTTAATGATGCTATAT	AAAGGTGAAATTAATTTTTAAAGTCA

Supplementary Table 2. Sequences of shRNA/siRNA for specific genes knockdown

Target genes	Sense sequence (5'-3')	Antisense sequence (5'-3')
Mouse *Stat6*	AGCAGGAAGAACTCAAGTTTA	TAAACTTGAGTTCTTCCTGCT
Mouse *Trim24*	CCCAAGTTGGAGTCATTCGAT	ATCGAATGACTCCAACTTGGG
Mouse *CBP*	TAACTCTGGCCATAGCTTAAT	ATTAAGCTATGGCCAGAGTTA
Control (shRNA)	TTCTCCGAACGTGTCACGT	ACGTGACACGTTCGGAGAA
Human *TRIM24*	CCAUUUCUGUUAACCUCUUAU	AUAAGAGGUUAACAGAAAUGG
Control (siRNA)	UUCUCCGAACGUGUCACGUdTdT	ACGUGACACGUUCGGAGAAdTdT
