# Supplementary material for: Modulation of M2 macrophage polarization by the crosstalk between Stat6 and Trim24
Source: Nat Commun. 2019 Sep 25;10:4353. doi: 10.1038/s41467-019-12384-2 (PMC6761150; doi:10.1038/s41467-019-12384-2)
Supplement: Supplementary file 3 — Reporting Summary [file 41467_2019_12384_MOESM3_ESM.pdf]

nature research

Corresponding author(s): Yichuan Xiao

Last updated by author(s): Sep 2, 2019

Reporting Summary

Nature Research wishes to improve the reproducibility of the work that we publish. This form provides structure for consistency and transparency in reporting. For further information on Nature Research policies, see Authors & Referees and the Editorial Policy Checklist.

Statistics

For all statistical analyses, confirm that the following items are present in the figure legend, table legend, main text, or Methods section.

n/a	Confirmed	
☐	☒	The exact sample size (*n*) for each experimental group/condition, given as a discrete number and unit of measurement
☐	☒	A statement on whether measurements were taken from distinct samples or whether the same sample was measured repeatedly
☐	☒	The statistical test(s) used AND whether they are one- or two-sided<br>*Only common tests should be described solely by name; describe more complex techniques in the Methods section.*
☐	☒	A description of all covariates tested
☐	☒	A description of any assumptions or corrections, such as tests of normality and adjustment for multiple comparisons
☐	☒	A full description of the statistical parameters including central tendency (e.g. means) or other basic estimates (e.g. regression coefficient) AND variation (e.g. standard deviation) or associated estimates of uncertainty (e.g. confidence intervals)
☐	☒	For null hypothesis testing, the test statistic (e.g. *F*, *t*, *r*) with confidence intervals, effect sizes, degrees of freedom and *P* value noted<br>*Give P values as exact values whenever suitable.*
☒	☐	For Bayesian analysis, information on the choice of priors and Markov chain Monte Carlo settings
☒	☐	For hierarchical and complex designs, identification of the appropriate level for tests and full reporting of outcomes
☒	☐	Estimates of effect sizes (e.g. Cohen's *d*, Pearson's *r*), indicating how they were calculated

Our web collection on statistics for biologists contains articles on many of the points above.

Software and code

Policy information about availability of computer code

Data collection	The Flow cytometric data: Beckman Gallios, Aria II (BD Biosciences)<br>The RNA seq data: Illumina HiSeq 4000<br>The qRT-PCR data: QuantStudio 7 Flex Real-Time PCR System (Applied Biosystems)<br>The Mass Spectrometry data: Integrated Proteomics Pipeline (v 4.3)
Data analysis	Statistical analysis: GraphPad Prism (ver. 5)<br>Flow cytometric analysis: FlowJo (Version7.6.1 for Windows)<br>RNA seq data analysis: RSEM (RNA-Seq by Expectation-Maximization) Software Package, DAVID Bioinformatics Platform, Ingenuity Pathway Analysis (QIAGEN Bioinformatics) and GraphPad Prism (ver. 5)<br>qRT-PCR analysis: QuantStudio Real-Time PCR Software (v 1.1, Applied Biosystems)<br>Western Blot quantitation: ImageJ (Version1.52a, developed by NIH)

For manuscripts utilizing custom algorithms or software that are central to the research but not yet described in published literature, software must be made available to editors/reviewers. We strongly encourage code deposition in a community repository (e.g. GitHub). See the Nature Research guidelines for submitting code & software for further information.

Data

Policy information about availability of data

All manuscripts must include a data availability statement. This statement should provide the following information, where applicable:

- Accession codes, unique identifiers, or web links for publicly available datasets
- A list of figures that have associated raw data
- A description of any restrictions on data availability

The RNA-Sequencing and ChIP-sequencing data have been deposited into the Gene Expression Omnibus (accession code GSE116588, GSE116566, GSE134158 and GSE134167). The source data underlying for the main figures and supplementary figures are provided as a Source Data file, which is included in the submission. All

other data supporting the findings of this study are available from the corresponding author on reasonable request.

Field-specific reporting

Please select the one below that is the best fit for your research. If you are not sure, read the appropriate sections before making your selection.

☒ Life sciences ☐ Behavioural & social sciences ☐ Ecological, evolutionary & environmental sciences

For a reference copy of the document with all sections, see nature.com/documents/nr-reporting-summary-flat.pdf

Life sciences study design

All studies must disclose on these points even when the disclosure is negative.

Sample size	Sample sizes were chosen based on previous publications, which were sufficient for statistical analysis.
Data exclusions	No data were excluded from the analysis.
Replication	The experimental findings were reliably reproduced. The replication numbers were described in the corresponding figure legends.
Randomization	Animals were randomly allocated to each group as described in Methods.
Blinding	Data collection of most mouse tumor experiments were performed in a double blinding manner. Cellular and biochemical experiments were not performed in a blinding manner.

Reporting for specific materials, systems and methods

We require information from authors about some types of materials, experimental systems and methods used in many studies. Here, indicate whether each material, system or method listed is relevant to your study. If you are not sure if a list item applies to your research, read the appropriate section before selecting a response.

Materials & experimental systems

n/a	Involved in the study
☐	☒ Antibodies
☐	☒ Eukaryotic cell lines
☒	☐ Palaeontology
☐	☒ Animals and other organisms
☒	☐ Human research participants
☒	☐ Clinical data

Methods

n/a	Involved in the study
☐	☒ ChIP-seq
☐	☒ Flow cytometry
☒	☐ MRI-based neuroimaging

Antibodies

Antibodies used	Flow cytometric antibodies for mouse CD3 (145-2C11), CD4 (RM4-5), CD8a (53-6.7), CD11b (M1/70), CD11c (N418), CD16/CD32 (93), CD19 (1D3), CD25 (PC61.5), CD44 (IM7), CD45 (30-F11), CD62L (MEL-14), Foxp3 (FJK-16s), B220 (RA3-6B2), F4/80 (BM8), and Ly-6G/Ly6C (RB6-8C5) were from eBioscience. Antibodies for Trim24 (14208-1-AP) and GAPDH (60004-1-1G) were from Proteintech. Antibodies for Trim24 (C-4, sc-271266), Hsp60 (H-1, sc-13115), Lamin B (C-20, sc-6216), p65 (C-20, sc-372), IκBα (C-21, sc-371), p38 (H-147, sc-7149), Erk1 (K-23, sc-94), Stat6 (M-20, sc-981), Irf4 (M-17, sc-6059), Ub (P4D1, sc-8017), c-Myc (9E10, sc-40) and donkey anti-goat IgG (HRP, sc-2020) were from Santa Cruz. Antibodies for Arg1 (9819), Mrc1 (91992), Jnk (9252), Tbk1 (3013), CBP (7389), Stat6 (5397), Acetylated-Lysine (9441), Normal Rabbit IgG (2729), P-Jnk (4668), P-Stat6 (9361), P-Akt (2965, 4060), P-p65 (3033), P-IκBα (2859), P-p38 (9215), P-Stat1 (7649) and P-Tbk1 (5483) were from Cell Signaling. Antibodies for β-actin (A2228) and Flag (A8592) were from Sigma-Aldrich. Antibody for HA (2013819) was from Roche. Antibodies for Lys48-Ubiquitin (05-1307) and Lys63-Ubiquitin (05-1308) were from Merck. Antibodies for Alexa Fluor 594 or Alexa Fluor 488 conjugated rabbit IgG (A21207, A11034), and Alexa Fluor Plus 488 conjugated Mouse IgG (A32723) were from Thermo Fisher Scientific. Antibody for mouse Lys383-acetylated Stat6 was generated from Shanghai Genomics.
Validation	The generated anti-Lys383-acetylated Stat6 antibody was validated in Fig. 2a-2c. The Stat6 (5397, Cell Signaling) antibody for ChIP-seq was validated by Active Motif. Other commercial antibodies have been validated by manufactures and the statements can be found on the manufactures' websites.

Eukaryotic cell lines

Policy information about cell lines

Cell line source(s)	HEK293T cells were provided by the laboratory of J. Qin (Shanghai Institute of Nutrition and Health). B16-F10 and MC38 cells

Cell line source(s)	were provided by the laboratory of Q. Zou (Shanghai Institute of Immunology). THP-1 cells was purchased from Cell bank of Chinese Academy of Sciences.
Authentication	None of the cell lines have been authenticated.
Mycoplasma contamination	All cell lines were tested negative for mycoplasma contamination.
Commonly misidentified lines (See ICLAC register)	No commonly misidentified cell lines were used.

Animals and other organisms

Policy information about studies involving animals; ARRIVE guidelines recommended for reporting animal research

Laboratory animals	Trim24 floxed mice were generated from Shanghai Research Center for Model Organisms, and then were backcrossed with C57BL/6 mice for at least for 6 generation. The C57BL/6 background Trim24 floxed mice were crossed with lysozyme M (LysM)-Cre mice to produce myeloid cell–conditional Trim24 knockout mice (Trim24flox/flox LysM-Cre; termed Trim24M-/-). Stat6+/- mice under C57BL/6 background (SJ-002828) were purchased from Shanghai Research Center for Model Organisms and were intercrossed to generate Stat6-/- (KO) and Stat6+/+ (wild-type, WT) mice, which were used in the experiments.
Wild animals	NA
Field-collected samples	NA
Ethics oversight	All mice were maintained in a specific-pathogen-free facility, and all animal experiments were complied with all relevant ethical regulations for animal testing and research, and were in accordance with protocols approved by the institutional Biomedical Research Ethics Committee, Shanghai Institutes for Biological Sciences, Chinese Academy of Sciences.

Note that full information on the approval of the study protocol must also be provided in the manuscript.

ChIP-seq

Data deposition

☒ Confirm that both raw and final processed data have been deposited in a public database such as GEO.

☒ Confirm that you have deposited or provided access to graph files (e.g. BED files) for the called peaks.

Data access links<br>*May remain private before publication.*	https://www.ncbi.nlm.nih.gov/geo/query/acc.cgi?acc=GSE134167
Files in database submission	1. Metadata spreadsheet: ChIP-seq of WT and Trim24-KO PM.xls;<br>2. Processed data files: WT-IL-4_ChIPSeq.bw, Trim24-KO-IL-4_CchIPSeq.bw, Input_DNA.bw;<br>3. Raw data files: WT-IL-4_ChIPSeq.fastq, Trim24-KO-IL-4_ChIPSeq.fastq, Input DNA.fastq.
Genome browser session (e.g. UCSC)	No longer applicable.

Methodology

Replicates	ChIP-seq for each group of the cells, which were pooled from two mice, was performed once.
Sequencing depth	The sequencing procedure was performed with llumina NextSeq 500. The total and uniquely mapped reads were listed below:<br>1. WT-IL-4_ChIPSeq: 46856377 reads total, 20751024 uniquely mapped, 75bp, single-end;<br>2. Trim24-KO-IL-4_ChIPSeq: 66422732 reads total, 15210169 uniquely mapped, 75bp, single-end;<br>3. Input_DNA: 52458698 reads total, 37823894 uniquely mapped, 75bp, single-end.
Antibodies	Stat6 (D3H4, 5397, Cell Signaling)
Peak calling parameters	The main peak caller used at Active Motif is MACS (Zhang et al., Genome Biology 2008, 9:R137).
Data quality	The Stat6 (D3H4, 5397, Cell Signaling) antibody for ChIP-seq was validated by Active Motif. The correct size of chromatin and libraries was checked in a gel. Only raw reads passing the QC were used for alignment. Tracks for each ChIP were generated and inspected in order to check the quality of the data.
Software	The average profile for tag distributions was generated using deeptools2. Visualization was performed in UCSC Genome Browser.

Flow Cytometry

Plots

Confirm that:

☒ The axis labels state the marker and fluorochrome used (e.g. CD4-FITC).

☒ The axis scales are clearly visible. Include numbers along axes only for bottom left plot of group (a 'group' is an analysis of identical markers).

☒ All plots are contour plots with outliers or pseudocolor plots.

☒ A numerical value for number of cells or percentage (with statistics) is provided.

Methodology

Sample preparation	The preparation of human or mouse primary macrophages were described in Methods. Isolation of tumor infiltrating immune cells from mouse and human cancer samples were described in Methods.<br>For surface staining, cells were harvested and washed by PBS for 2 times, then stained in PBS containing 0.1% w/v bovine serum albumin for 45 minutes at 4 degree, followed by washing by PBS for 2 times prior to Flow Cytometry analysis.<br>For intracellular staining of mouse primary T cells to measure cytokine secretion, cells were harvested and washed for 2 times with PBS, fixed with 4% PFA for 12 minutes at room temperature and permeated with Permeabilization Buffer containing 0.1% Triton X100 and 0.5% w/v bovine serum albumin in PBS, after which the cells were intracellularly stained in Permeabilization Buffer with antibodies for at least 2 hours at 4 degree. Stained cells were washed 2 times with PBS prior to Flow Cytometry analysis.
Instrument	The Flow cytometric data were collected by Beckman Gallios or BD Aria II flow cytometer.
Software	Data were analysed with FlowJo (Version7.6.1 for Windows).
Cell population abundance	The tumor-associated macrophages (Figure 7h-j) were sorted by BD Aria II, and the purity was higher than 95% as determined by CD11b and F4/80 double positive population.
Gating strategy	For all experiments, cells were first gated by FSC/SSC to exclude debris, followed by gating FSC-A and FSC-H to eliminate nonsinglets. Then, target cell population for further analysis were gated by cell surface marker. The detailed gating strategies for all the flow cytometric analysis were described and showed in Supplementary Figure 9.

☒ Tick this box to confirm that a figure exemplifying the gating strategy is provided in the Supplementary Information.
